# Wildfire Early Warning System Based on a Smart CO_2_ Sensors Network

**DOI:** 10.3390/s25072012

**Published:** 2025-03-23

**Authors:** Alessio De Rango, Luca Furnari, Fabio Cortale, Alfonso Senatore, Giuseppe Mendicino

**Affiliations:** Department of Environmental Engineering, University of Calabria, Rende, 87036 Cosenza, Italy; luca.furnari@unical.it (L.F.); fabio.cortale@unical.it (F.C.); alfonso.senatore@unical.it (A.S.); giuseppe.mendicino@unical.it (G.M.)

**Keywords:** CO_2_ sensors, LSTM, AutoEncoders, early warning system, wireless sensors network, wildfire detection

## Abstract

Climate change exacerbates wildfire risks in regions like the Mediterranean, where rising temperatures and prolonged droughts create ideal fire conditions. Adapting to this scenario requires implementing advanced risk management strategies that leverage cutting-edge technologies. Wildfire early warning systems are crucial tools for detecting fires at an early stage, helping prevent potential future damage. This paper proposes a smart CO_2_ sensor network-based early warning system, relying on a platform that enables the connection, management, and processing of data from the devices through the cloud. The wildfire early warning system was tested in a real controlled experiment, in which 44 sensors were deployed in strategically selected locations at varying distances from the fire. To enhance early detection, three Artificial Intelligence (AI) models were developed using AutoEncoders (AEs) and Long-Short-Term Memory (LSTM), and these were compared to a simple threshold-based (NO-AI) model. All AI models, especially the LSTM-based model, were able to extract more valuable information from the CO_2_ records, activating up to 56% more sensors than the NO-AI model in less time and tracking potential fire front propagation based on wind patterns. Therefore, the system not only improves early fire detection models but also effectively supports firefighting operations.

## 1. Introduction

Forest fires are a complex phenomenon that affects many ecosystems and can cause several issues for human life and activities [1]. Many factors, including climate, fuel type, and human presence, influence the number and extent of such fires [2]. Climate change cannot be omitted among the factors triggering or exacerbating this phenomenon, mainly due to the increasing anthropogenic pressure on wildland ecosystems [3]. Unsurprisingly, it is estimated that 96% of fires in Europe are of anthropogenic origin, either intentional or due to negligence [4,5,6,7,8].

Detecting wildfire ignitions early is crucial to reducing its impacts, as it enables timely intervention before fires escalate beyond control. Quantifying such aspects is highly challenging because of the large number of factors to take into account. However, some studies show that most damage and fatalities occur in the first 4 h after ignition [9]. With the intensification of climate change, wildfires are occurring more frequently, covering larger areas and burning with greater intensity worldwide. This highlights the need for advanced and reliable early detection technologies. Among the first signs of a wildfire is smoke production. Smoke is a rich and complex mixture of particulate matter [10,11,12] and gases, including greenhouse gases such as CO_2_, CO, O_3_, and CH_4_. Of course, the specific composition of each smoke varies depending on the type of fuel and the microclimatic conditions in which the wildfire occurs. Still, the above gases are among the most common [13].

From an early detection system point of view, a non-trivial problem is the propagation of these gases in the atmosphere, highly influenced by numerous external factors. For this reason, data-driven systems, increasingly based on AI (Artificial Intelligence), are preferred to physically based models, which require a lot of spatially distributed meteorological data [14]. AI has proven useful in early warning system monitoring and development. Several studies in the literature demonstrate its effectiveness [15,16,17,18,19,20,21]. In recent years, various sorts of data sources have been employed for fire detection, such as data from aerial (satellites or drones) and ground-based (cameras) remote sensing, as well as data from in situ sensor networks.

Table 1 summarises some recent contributions to the literature that prove the effectiveness of the AI methodology as compared to different data sources. Ref. [15] aimed to assess hardware accelerators’ efficiency in edge computing for real-time wildfire alerts, leveraging Convolutional Neural Networks (CNNs) and hyperspectral data analysis. Wildfires in Australia were analysed as a practical case study using data obtained from the PRISMA (PRecursore IperSpettrale della Missione Applicativa) satellite. In [16], Artificial Neural Networks and Support Vector Machines (SVMs) were used to process satellite imagery from vast regions, enabling the prediction of wildfire occurrences. Ref. [17] introduced the Flame and Smoke Detection Dataset (FASDD), a groundbreaking collection of over 120,000 diverse images depicting various fire scenarios aimed at advancing fire detection models. This dataset was utilized to evaluate Swin Transformer models, which exhibited commendable fire detection performance. Focusing on the combined use of ground-based remote sensing (images acquired from optical cameras) and sensors, ref. [18] provided a CNN methodology that leverages transfer learning alongside data augmentation techniques. In [19], Inception-v3, a CNN-based transfer learning approach, was proposed by incorporating Radial Basis Function Networks (RBFNs) along with Rapid and Accurate Image Super-Resolution (RAISR). In [20], a wireless sensor network (WSN) was employed to develop early-warning systems with high accuracy. Sensors (i.e., optical cameras) in WSN collected remotely sensed images from the target environment, while deep learning (DL) methods were used to analyse the collected images to detect wildfires. Ref. [21] presented a study in which the application of AI relies only on a point sensor network monitoring several meteorological and air quality (PM_2.5_, PM_10_, CO, and NO_2_) data. More specifically, a U-Convolutional Long Short-Term Memory (ULSTM) neural network was developed to extract key spatial and temporal features from the dataset to address the spatial and temporal nature of the location of wildfire evolution.

Indeed, within a flexible and reliable combined approach, based on both images and point sensors, the latter represent a key element in an early warning system because they provide the ground truth, quantitatively providing information about the time-space evolution of wildfire intensity and its effects on the surrounding environmental features. Many portable sensors, particularly gas and particle analysers, have been developed for this aim. Since a great number of these sensors should be deployed in the field in an operational context, low-cost devices are particularly desired. In [22], a thorough evaluation of low-cost CO_2_ sensors across various price ranges is conducted, comparing their performance with a reference-grade instrument and exploring the potential for calibration through different machine learning techniques. In [23], the suitability and accuracy of three commercially available air quality sensors were examined through controlled laboratory experiments, highlighting the extent to which low-cost, portable emission sensors can be effectively used for wildfire measurements in the field. In [24], a compact and sensitive dual-gas laser absorption sensor was created for detecting smoldering peat fires through the real-time monitoring of transient CO_2_ and CH_4_ emissions. In [25], 13 Outdoor Aerosol Samplers (OASs) were deployed around a large prescribed fire in southern Colorado to evaluate their effectiveness as a smoke monitoring tool.

Within the framework of an integrated (joint ground-based images from camera systems and sensors) forest fire early warning system, this paper presents the design, implementation, and testing of a monitoring network composed of robust and versatile moderate-cost CO_2_ sensors, whose measurements were treated using AI-based techniques to extract early wildfire detection information. The sensors utilized were the Milesight EM500-CO_2_ connected by a UG67 Gateway. This system permits the design of a network of CO_2_ sensors utilizing LoRaWAN technology. The network was evaluated in a field experiment to determine its reliability and accuracy, positioning dozens of CO_2_ sensors at various distances (in the order of 10^1^ m) from a controlled fire. A platform was created that enabled the collection, storage, and display of data from sensors during the experiment via a remote service. Furthermore, three Artificial Intelligence models based on an AutoEncoder and LSTM were built to allow autonomous fire detection. These models were compared to a traditional threshold alerting strategy that does not involve artificial intelligence.

The paper is structured as follows: Section 2, regarding the materials and methods, describes the CO_2_ sensors used, the field experiment performed, the architecture of the data acquisition system, the AI methodology, the alert threshold strategies, and the dataset. Section 3 presents and discusses the results achieved and highlights the ability of the AI-based models to codify information from network measurements to enhance the early warning capacity of the system. Finally, Section 4 summarize the main features and limits of the system and delineate future outlooks.

## 2. Materials and Methods

### 2.1. Sensors Description

**Figure 1:**
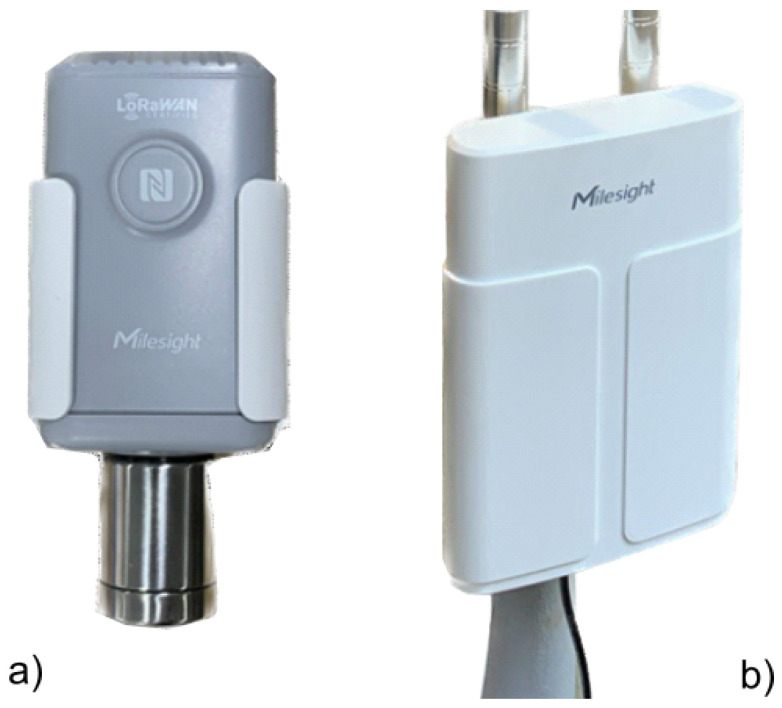
(**a**) A Milesight sensor EM500-CO_2_; (**b**) A Milesight UG 67 Gateway.

The Milesight EM500-CO_2_ is a sensor that measures not only the concentration of carbon dioxide (CO_2_) but also various environmental parameters such as temperature, humidity, and barometric pressure under external or extreme conditions. The Milesight EM500-CO_2_ is considered a moderate-cost sensor [26]. This equipment uses LoRaWAN technology to transmit data. It is a low-power sensor and lasts for many years. Figure 1a shows the Milesight EM500-CO_2_, and Figure 1b depicts the Milesight gateway UG67. Equipped with the LoRa SX1302 chip and a 1.5 GHz 64-bit quad-core processor, the UG67 can handle connections with more than 2000 devices, providing coverage up to 15 km in ideal conditions and about 2 km in urbanised environments [27].

The Milesight EM500-CO_2_ is composed of an integrated carbon dioxide sensor, which is a non-dispersive infrared type sensor that is well known for its high accuracy and reliability in measuring gas concentrations. The working principle of the NDIR sensor involves the passage of infrared light through a chamber containing an air sample. CO_2_ molecules absorb some specific wavelengths of this light. The sensor uses a passive air sampling mechanism, in which air naturally diffuses into the sensor’s measurement chamber without the assistance of pumps or fans. Such a sensor is less reactive than an active flow sensor, but it makes the system cheaper and easier to maintain. A detector measures how much light is passing through the chamber, so the amount of light absorbed indicates the concentration of CO_2_. The method gives an accurate measurement ranging between 400 ppm and 5000 ppm, with an accuracy of ±(30 ppm + 3% of reading) from 0 °C to 50 °C, under relative humidity from 0 to 85%. From now on in this paper, for the sake of simplicity, we will refer to CO_2_ concentration measurements as CO_2_ measurements.

Along with the CO_2_ sensor, the EM500-CO_2_ now includes MEMS sensors for temperature, relative humidity, and barometric pressure measurements. These sensors are not original devices but are miniaturized through the use of mechanical and electronic components, in addition to being very sensitive and offering fast responses. The temperature sensor is used within a total range from −30 °C to +70 °C, with an accuracy of ±0.3 °C from 0 °C to +70 °C and ±0.6 °C from −30 °C to 0 °C. The humidity sensor works under a complete range from 0% to 100% relative humidity and ±3% accuracy between 10% and 90% RH and ±5% outside that range. The sensor for barometric pressure can measure from 300 to 1100 hPa with precision ±1 hPa. Although the sensor can measure several environmental variables, in this work, only CO_2_ is taken into consideration. EM500-CO_2_ is encased in an IP65-rated enclosure, which protects it from dust and water jets [28,29].

### 2.2. Description of the Experiment

**Figure 2:**
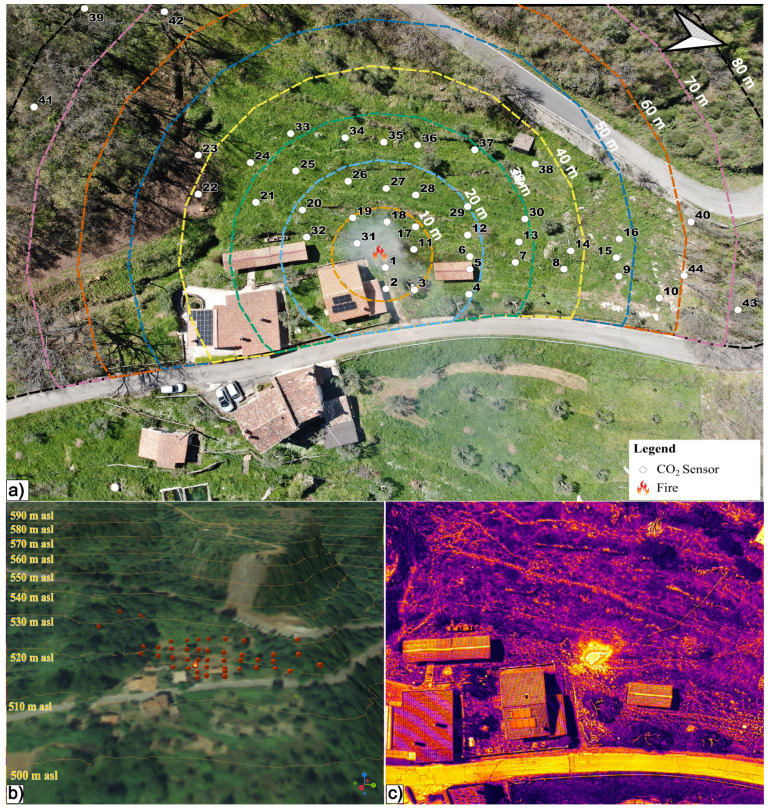
(**a**) Overview of the study area where 44 CO_2_ sensors were located. The number identifies the sensors, and the colored dashed circles indicate the equidistant lines with respect to the ignition point (flame symbol). (**b**) 3D view of the study area (z factor = 1.5) with the 44 sensors (red dots), the ignition point, and the contour lines with 10 m spacing (thin yellow lines). (**c**) Thermal image recorded by the drone during the fire experiment.

The CO_2_ sensors were placed in the morning of 23 March 2024, creating a regular grid near the trigger point as much as possible. In addition, 6 sensors were placed at the extremes of the investigated hillslope, at longer distances and higher elevations. All sensors were placed on small poles at a height of 1.5 m above the ground, and the measurement of the temporal resolution of the sensor was set to two minutes. To evaluate the system’s ability to detect wildfires, we lit a controlled fire on 28 March 2024, 5 days after the sensor’s installation, from 11:28 to 13:18 (local time) in an area dominated by olive groves in the Dipignano (CS) Municipality (southern Italy). Figure 2a shows the location of the 44 sensors with respect to the trigger point, starting from which equidistant lines are highlighted. Each sensor was labeled with a corresponding number to identify it during the experiment. Figure 2b highlights the elevation that spans from approximately 500 m asl to 530 m asl at the locations of sensors 39–42 (i.e., the two farthest).

As a preliminary step, we installed a weather station to monitor the wind speed and direction in real time, two key factors influencing plume air propagation. The station, a model of the Raddy WF-100C type, allows the real-time monitoring of precipitation, wind direction and intensity, air temperature, relative humidity, atmospheric pressure, solar radiation, and UV index. A solar panel powers the sensors, and the station communicates data every five minutes in real time on online clouds to create a historical database. This comprehensive data collection enables researchers to analyze trends and make informed decisions regarding air quality and environmental impact. Furthermore, integrating this technology promotes a deeper understanding of atmospheric dynamics, ultimately aiding in developing more effective pollution control strategies.

During the fire experiment, 8 m^3^ of olive and oak tree branches, cut during the previous weeks, were used as fuel and burnt. In order to simulate a real fire, the branches were not cleared, leaving the twigs and leaves intact, so in the estimated volume there were many voids. No other fuel was used to simulate a real wildfire without any accelerators. The fire experiment started at 11:28 and ended at 13:18, when all the fuels were consumed.

To also monitor the temperature gradient during the fire experiment, we performed a UAV flight with a DJI 350RTK matrix equipped with a thermal camera, model DJI Zenmuse H20T. Figure 2c shows an example of a thermal image recorded during the fire experiment. Figure 2a shows, as background, the RGB image recorded from the UAV and highlights the smoke propagation in the atmosphere. In the Supplementary Material, we attached a video highlighting the temperature and RGB evolution near the ignition point during part of the field experiment.

### 2.3. Data Acquisition System

**Figure 3:**
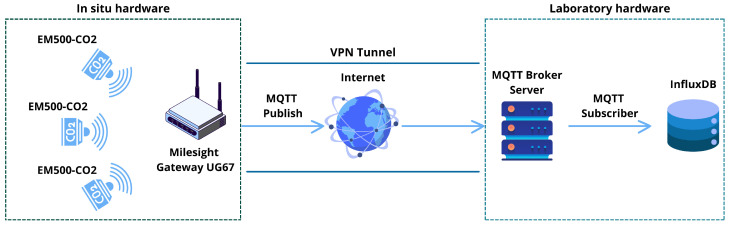
Representation of the system architecture.

With the aim of testing the sensor network in a real-world context, a data-gathering, storage, and visualization system was implemented. The data acquisition system was based on the MQTT (Message Queuing Telemetry Transport) protocol. An MQTT broker server was set up. With this protocol, it is possible to define a publisher (in the case of the experiment, the Milesight Gateway UG67) and a subscriber, i.e., a service by which whenever a sensor sends a data item, it is promptly received and stored within a database. The database used refers to InfluxDB, a versatile tool for storing time series, while services based on Apache NiFi were implemented to manage the entire data flow from the sensors. Moreover, a VPN tunnel was implemented for security reasons, allowing the milesight gateway to connect to the remote MQTT broker server. Figure 3 depicts the data acquisition system architecture.

### 2.4. Methodology

This research adopts two different kinds of AI techniques as follows: AutoEncoders (AEs) and Long-Short-Term Memory (LSTM) networks. Rumelhart established the notion of the AE in a research article in 1985 [30]. AEs are a kind of neural network used to learn and reconstruct input data (CO_2_ concentration values, in our case) based on unsupervised learning. The main goal of unsupervised learning is to obtain an “informative” data representation. AEs encode input data into a compressed and semantically understandable form and then accurately decode it to recover the original input data [31]. In more detail, AEs are neural networks that use the back-propagation technique to learn features. They do not need labeled data for training, so they are typically employed for unsupervised learning tasks. Because of this, AEs can be used in scenarios where labeled data are complex to come by or prohibitively expensive [31]. AEs learn important features from data automatically. Hence, they do not require human feature engineering. This saves a lot of preprocessing time and effort. The AEs encode essential features from the input data, resulting in meaningful representations of the data in the latent space.

On the other hand, LSTMs are recurrent neural networks. Generally, unlike standard feedforward neural networks, Recurrent Neural Networks (RNNs) feature connections that create directed cycles, allowing them to retain a memory of past inputs. RNNs are well suited to work with time series data and sequential dependencies. In particular, LSTM networks are a form of RNN that detects long-term dependencies in sequential data. Unlike traditional RNNs, LSTMs can efficiently remember critical information over lengthy time periods while avoiding problems like disappearing gradients. LSTMs are especially useful in feedforward prediction for time series analysis.

**Figure 4:**
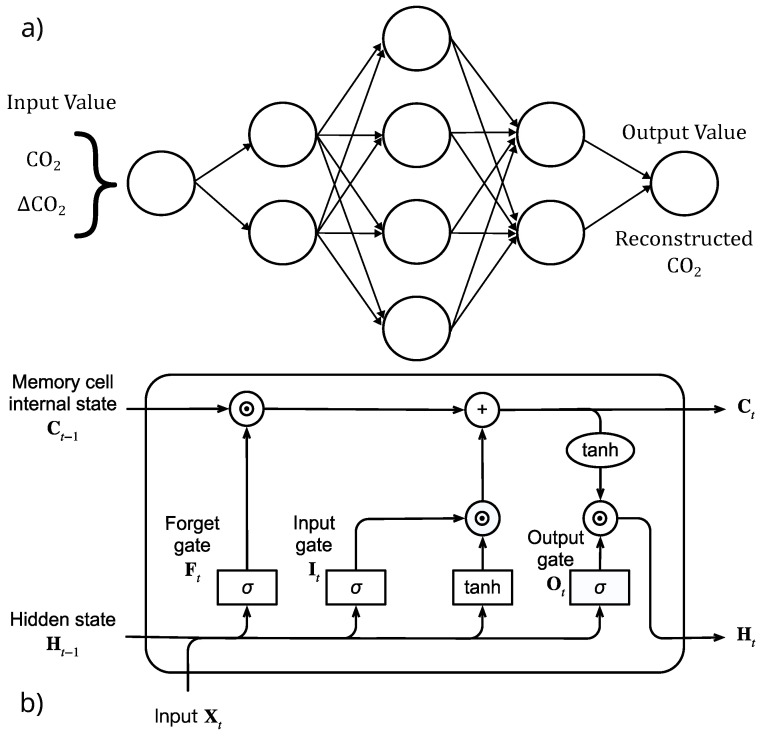
AutoEncoder (**a**) and LSTM (**b**) architectures.

The main goal of this study is to explore how artificial intelligence approaches might be utilized to automatically extract information from CO_2_ sensor networks to enhance early wildfire detection. The performance of AI models was compared to a classical threshold procedure that did not use AI techniques, called NO-AI hereafter. A total of three AI models were developed, implemented, and tested, two based on AE, such as AE CO_2_, AE ΔCO_2_, and one on LSTM, using as input data the CO_2_ time series recorded by the 44 sensors. Since each sensor provided a different CO_2_ concentration value at each time step, all three AI models were trained separately for each sensor. The three AI models are described below as follows:

- *AE CO_2_*: The objective was to train the model before the fire event to reconstruct the detected CO_2_ data efficiently. Once the model has been trained, a CO_2_ concentration anomaly triggers an error in the model’s reconstruction, resulting in an alarm. The model comprised three hidden layers, with 10, 20, and 10 neurons, respectively, achieved after several experiments. The best hyperparameters for the model are a batch size of 64, with a learning rate of 0.001 for a total of 50 epochs. The Adam optimizer was used with a Mean Square Error (MSE) loss function, and the ReLU activation function was adopted. Figure 4a illustrates the AE architecture.
- *AE* Δ*CO_2_*: The same methodology applied to the AE CO_2_ was used. The main difference refers to the input data, since the model learns to reconstruct the ΔCO_2_ change between the value that was detected at time *t* and that at time t−1. The development of this model was prompted by the observation that the CO_2_ recorded by the sensors exhibits a highly oscillatory pattern rather than a linear trend. The model used the same hyperparameter as the *AE CO_2_* model. Nevertheless, the best architecture adopted comprised 5 hidden layers, with 10, 20, 30, 20, and 10 neurons, respectively. Figure 4a illustrates the AE architecture.
- *LSTM*: The best LSTM model developed was trained by considering a time window of 10 consequent measurements with the prediction of the next CO_2_ value and configured with 32 memory units. A total of 50 epochs with a batch size of 64 were used. The Adam optimizer was also considered with the MSE loss function and a learning rate of 0.001. Figure 4b illustrates the LSTM architecture.

The models were developed using the Keras and TensorFlow deep learning API written in Python3. Moreover, dataframe datastructure and numpy library were used to manipulate the input data of the models. All AI computational analyses were performed on a scientific workstation equipped with an AMD Ryzer 5 5600H and an NVIDIA GeForce RTX 3050 GPU.

### 2.5. Alert Threshold

An alert threshold was considered since the developed AI models do not automatically discriminate the anomalies. To calibrate the alert thresholds for all the models, a calibration algorithm was designed to find the best value that discriminates from a CO_2_ usual trend to an anomaly/alert. The calibration algorithm considered the training period in which different thresholds were tested to avoid false alarms. For each of the 44 sensors, different thresholds were considered for each model. The system raises an alert if the AI model error-reconstructed value differs with respect to the observed CO_2_ more than the calculated threshold. Equation (1) explains the alert mechanism.

(1) $$f\left(O_{i}^{t}\right)=\left\{\begin{array}{cc} alert & \mathrm{if}\;O_{i}^{t}>=M_{i,m}^{t}+\Delta_{i,m}\\ no\_alert & \mathrm{if}\;O_{i}^{t}<M_{i,m}^{t}+\Delta_{i,m} \end{array}\right.$$

where $O_{i}^{t}$ identifies the observed CO_2_ value related to the sensor *i* at time *t*, $M_{i,m}^{t}$ depicts the modeled CO_2_ value for the sensor *i* and model *m*, and $\Delta_{i,m}$ is the alert threshold calibrated for every sensor and model, with the intention to avoid false alarms during the training period.

The NO-AI methodology considers the maximum value of individual sensors in the training period (i.e., 5 days before the fire event) as the alert threshold. This threshold allows to avoid false alarms during the training period and achieve the best performance during the test period.

### 2.6. Dataset

The AI models were trained on a dataset composed of the time series of the CO_2_ concentrations recorded from 5 days before the fire experiment in normal (i.e., undisturbed) conditions. The CO_2_ sensors were configured to measure CO_2_ every two minutes. A total of 3521 CO_2_ values for each sensor were registered. In particular, 80% was used for training and 20% for validation (2817 and 704 measurements, respectively).

A test dataset composed of measurements from early morning to the end of the wildfire event was considered to evaluate the performance of the AI models. It is worth noting that the test set takes into account not only the fire event but also undisturbed CO_2_ conditions, therefore, also checking for the possibility of false alarms. The test dataset is composed of a total of 236 measurements for each sensor.

## 3. Results and Discussion

**Figure 5:**
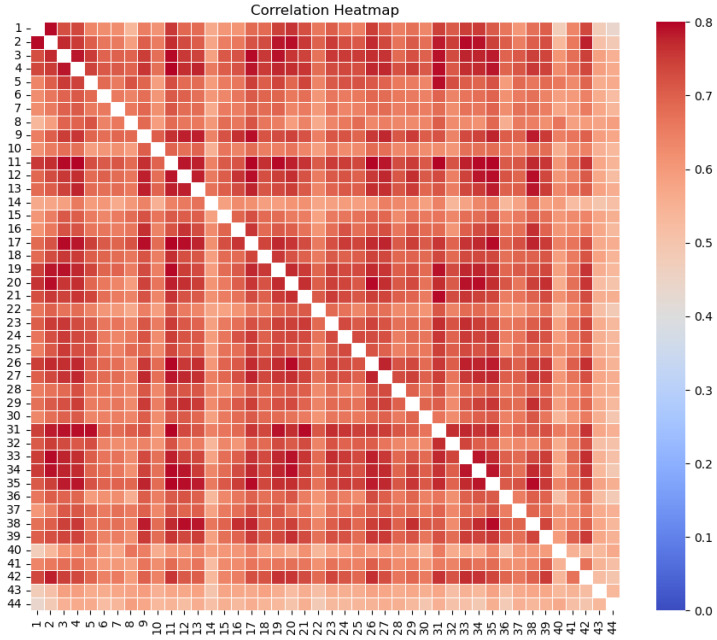
Correlation heatmap considering the CO_2_ concentration in the 44 sensors installed. The correlation was calculated during the 5 days before the fire experiment period, used to train the AI models and select the NO-AI thresholds.

Figure 5 shows a Pearson correlation heatmap of the CO_2_ concentration time series recorded by all 44 sensors in the no-fire period with a 2 min time resolution. It can be immediately observed that correlations were high during this period. Moreover, all correlations were significant at a 95% level. Fifteen sensors, mainly placed in the middle of the network, showed average correlations with all other sensors higher than 0.7 (with a maximum average correlation of 0.73 for sensor 11). The three sensors with the lowest average correlation with the others (lower than 0.6) were 40, 43, and 44, i.e., the three sensors positioned further north. Such results are expected given the short distance among the sensors measuring almost simultaneously several diurnal–nocturnal CO_2_ cycles. The same analysis performed for the much shorter fire period (only 110 min) led to much lower correlation values (not shown) because of the effects induced by the fire on the atmosphere, the local variability of the measurements, and the instruments’ accuracy, making the correlations not significant, even for sensors close to each other and not affected by fires.

**Figure 6:**
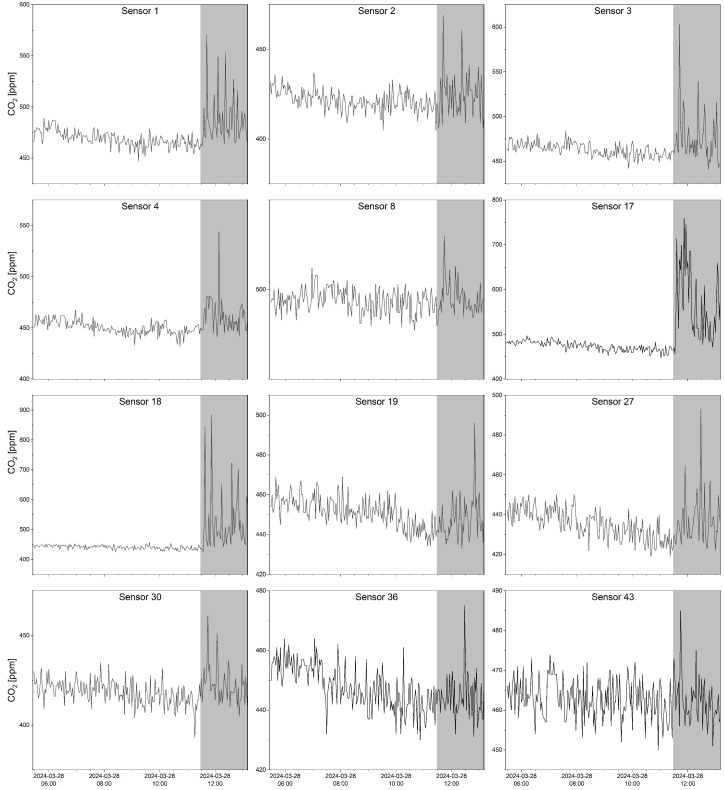
A subset of CO_2_ time series related to 12 sensors recorded on 28 March 2024 from early morning until the wildfire event. The gray area indicates the fire experiment period.

Figure 6 graphically illustrates the anomalies of the CO_2_ concentration time series recorded the morning of the experiment, with the gray area highlighting the fire experiment time. The downwind sensors exhibited high sensitivity to the presence of fire. For example, sensors 17, 3, and 18, positioned between 5 and 10 meters from the fire, recorded several significant spikes in CO_2_ levels, strongly modifying the expected trend before the fire experiment. Similar patterns, though with fewer spikes, were observed with sensors located further from the fire, such as sensors 4, 8, and 43, positioned at distances of 20, 30, and 70 m, respectively, (Figure 2a). However, a more comprehensive statistical analysis (Table A1 in the Appendix A) highlighted that, extending the analysis period backward to the date of installation of the sensor network, only in 16 cases out of 44 the maximum CO_2_ concentration values recorded during the fire period were higher than the no-fire period. Furthermore, 13 out of these 16 stations were located at less than 20 m from the controlled fire, and only in 6 cases the CO_2_ maxima were more than 10% higher than the no-fire period. Such an analysis demonstrates that detecting a clear fire signal in progress through oversimplified threshold-based rules could be ambiguous or ineffective, especially for sensors not very close to the fire. Unconventional methods such as AI-based models could be particularly helpful in extracting more valuable and timely information from the recorded time series.

The effectiveness of the models in identifying the spikes is depicted in Table 2, showing the total number of alerts the models provided for all sensors with at least one alert. The total number of sensors for which at least one methodology (i.e., LSTM) produced an alert was 26 out of a total of 44 sensors used. The sensors with the most alerts were 17 and 11, with a total of 89 alerts considering all four models, and a maximum of 36 alerts provided by the NO-AI model, while those with the fewest alerts were 5, 29, and 44, with only one alert provided. In several cases, only one model provided alerts; more specifically, LSTM was the only model to provide alerts in sensors 5, 29, and 44. The total number of alerts provided by NO-AI was 127, followed by LSTM with 101, AE ΔCO_2_ with 97, and finally, AE CO_2_ with 93 total alerts. The maximum calibrated thresholds $\Delta_{i,m}$ retrieved by following the methodology explained in Section 2.5 are 33.1, 55, and 30.9 ppm for the AE CO_2_, AE ΔCO_2_, and LSTM models, achieved on sensors 40, 32, and 16, respectively. Considering all 44 sensors, the model with the lowest median threshold is the LSTM with 22.4 ppm, compared to 23.4 ppm for AE CO_2_ and 30.5 ppm for AE ΔCO_2_. These differences could be due to the different input information used by the models. More specifically, AE CO_2_ and LSTM directly use the measured CO_2_ concentration values, whereas AE ΔCO_2_ relies on the difference between two consecutive measurements. Moreover, the median threshold of AE ΔCO_2_ is approximately within the sensor’s declared accuracy range of ±30 ppm.

**Figure 7:**
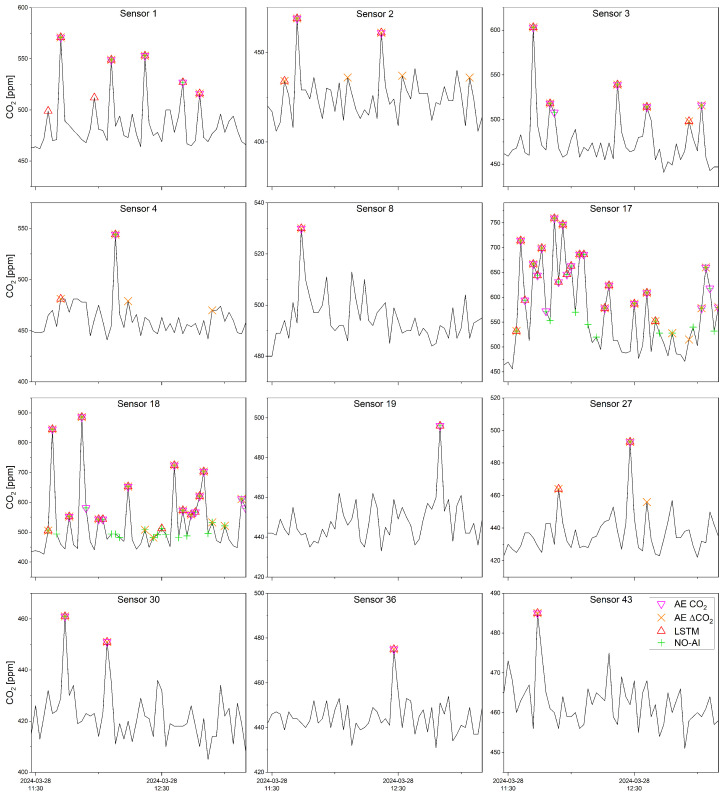
Subset of CO_2_ time series related to 12 sensors, recorded during the wildfire experiment and compared with the alerts detected by the 4 different models.

In more detail, Figure 7 shows the temporal evolution of the CO_2_ concentration only during the fire experiment (i.e., from fire lighting to extinguishing) and highlights the alerts provided by the different models. On the one hand, some sensors, according to which the alert was not triggered following the NO-AI model, were instead alerted following the LSTM model (for example, sensors 8 and 36). More specifically, the anomaly in the CO_2_ signal was not such as to exceed the maximum value recorded in the previous five days. However, using AI predictive techniques, the difference between observed and predicted data was enough to trigger alerts. On the other hand, in sensors that recorded very high anomalies during the fire experiment, the NO-AI models provided alerts almost continuously (e.g., sensor 17 or 18).

**Figure 8:**
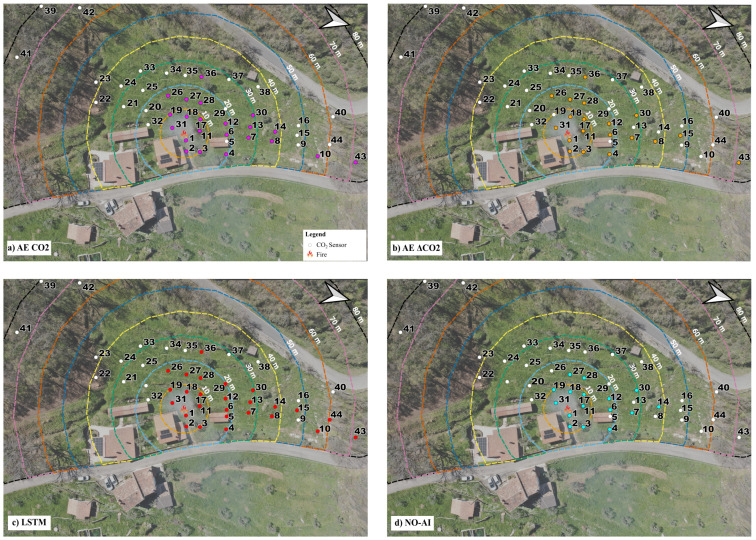
Spatial distribution of the sensors alerted during the fire experiment by the 4 different models: (**a**) AutoEncoder directly applied on the CO_2_ concentration; (**b**) AutoEncoder applied on the difference of CO_2_ concentration; (**c**) LSTM; and (**d**) NO-AI model.

Figure 8 illustrates the spatial distribution of the alerted sensors at the end of the fire experiment provided by the four models. The total number of alerted sensors was 22, 19, 26, and 16, achieved by the AE CO_2_, AE ΔCO_2_, LSTM, and NO-AI model, respectively. The NO-AI model not only alerted the lowest number of sensors, demonstrating to be the less sensible to changing CO_2_ conditions, but was also unable to alert sensors beyond a distance of 40 m, at which the CO_2_ concentration propagation was less evident by looking directly at the time series data. All the other models successfully alerted one of the furthest sensors (i.e., sensor 43). Moreover, LSTM was the only model to alert sensors 44 and 5, almost aligned in the same direction as sensor 43.

**Figure 9:**
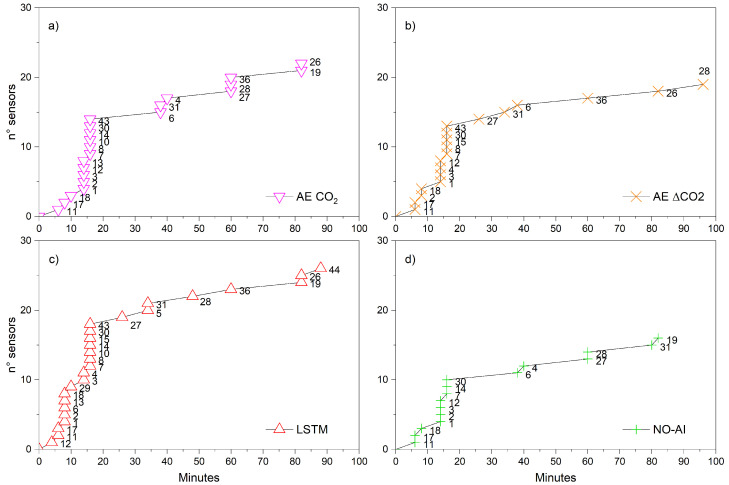
Temporal distribution of the alert provided by the different models since the beginning of the fire experiment. The x-axis indicates the minutes after the ignition, while the y-axis indicates the total number of sensors alerted. The label highlights the sensor ID: (**a**) AutoEncoder directly applied on the CO_2_ concentration; (**b**) AutoEncoder applied on the difference Δ of CO_2_ concentration; (**c**) LSTM; and (**d**) NO-AI model.

Figure 9 shows the number of sensors alerted and the timing of the first alert provided by all models from the fire ignition time. Such an analysis unambiguously reveals the efficiency of each model. Indeed, the NO-AI model is not only the one with the fewest alerted sensors, but it also has a large proportion of them presented as delayed first alerts with respect to all the AI-based methodologies. Considering the number of sensors alerted, the best model was the LSTM model, with nine sensors alerted within the first 10 min, while the NO-AI model only provided alerts to three sensors. After 20 min from the beginning of the field experiment, 18 sensors were alerted by the LSTM, 14 by the AE CO_2_, 13 by the AE ΔCO_2_, and 10 by the NO-AI model. The sensor for which the LSTM model performed best was sensor 6, where the LSTM-driven alert was advanced by 30 min compared to the NO-AI model.

Finally, Table 3 further highlights the capability of the LSTM model to detect alerts timely. Particularly, it shows the ratio between the Euclidean distance of each sensor from the ignition point to the time interval provided by the model for the first alert, therefore calculating a kind of celerity in providing alerts. In fact, given a fixed distance, higher values indicate that less time is needed by the model to alert the sensor. Table 3 shows that for many sensors the alerts were provided simultaneously by all models. However, in all cases in which a single model could be detected as the most timely, that was the LSTM model.

**Figure 10:**
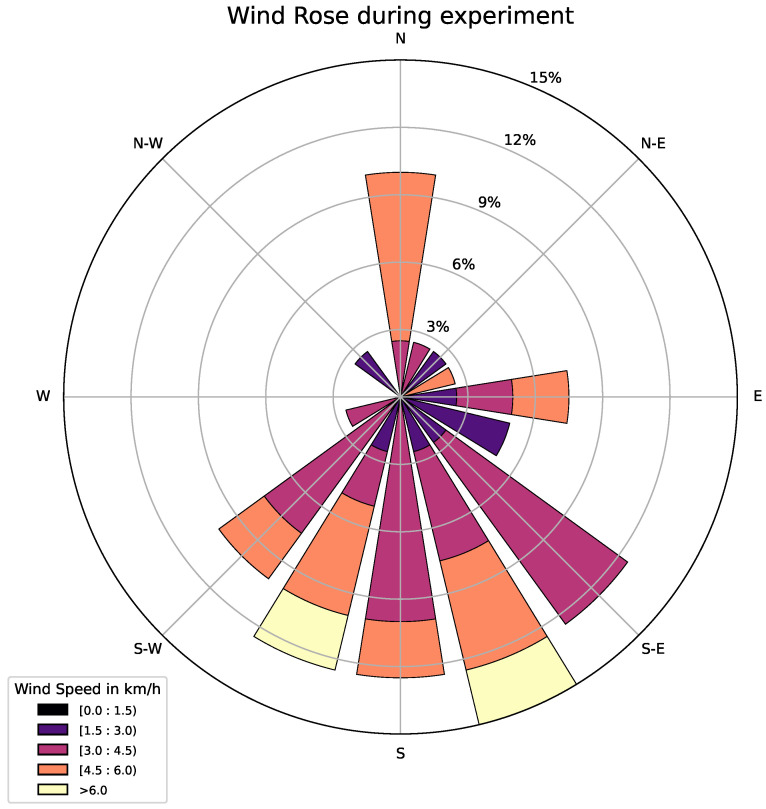
Wind rose obtained during the fire experiment. The color represents the wind speed in km/h, while the band length represents the percentage frequency. The angle represents the source direction of the wind.

As highlighted during the fire experiment, atmospheric conditions, particularly wind direction, are crucial for determining the evolution of the fire and, therefore, alerting the sensors. The prevailing wind direction during the fire experiment was from the south, as depicted in Figure 10, showing the wind rose frequency analysis during the experiment. The wind blew mainly, but not exclusively, from southerly directions, and never exceeded 6.4 km/h. Most of the sensors that detected the fires were in the north sector, aligned with the prevailing wind direction from the south.

**Figure 11:**
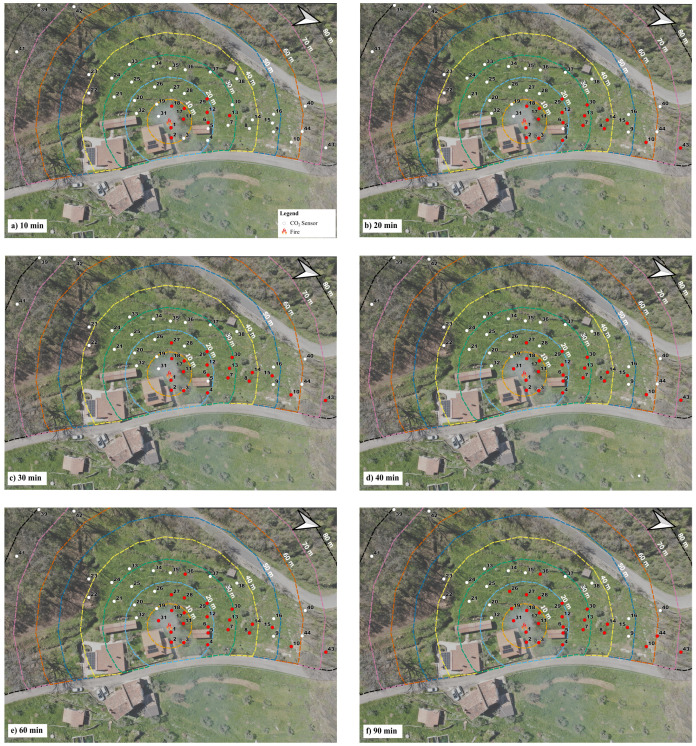
Spatial distribution of the sensors alerted after the ignition at: (**a**) 10, (**b**) 20, (**c**) 30, (**d**) 40, (**e**) 60, and (**f**) 80 min by adopting the best model (i.e., LSTM). The red dots indicate the alerted sensors and the white dots indicate the sensors not alerted.

To highlight those aspects, Figure 11 depicts the spatial distribution of the first anomaly detection related to the best model, the LSTM model, from the beginning of the fire event until the end. The red dots indicate where the model identified an alert. It highlights how, after approximately 40 min, the wind started blowing from another direction, further activating other sensors. The LSTM model could detect the fire within the first 10 min with 9 sensors (Figure 11a), and within the first 20 min with 18 sensors, but still limited to the north direction (Figure 11b). Then, more sensors were activated in the west direction, as evidenced, especially after 60 and 90 min (Figure 11e,f).

The key advantage of AI models, and in particular LSTM, lies in their ability to detect CO_2_ anomalies and generate alerts for the sensors that have actually captured such signals (i.e., the downwind sensors). The capability of capturing and enhancing even weak signatures of the wildfire effects provides added value to the measurements, leading to fewer missed alarms. From this point of view, a crucial role is played by the CO_2_ sensors, which should be as responsive and accurate as possible. In our case study, characterized by the adoption of passive and moderate-cost sensors, results, though satisfying in terms of alerting capacity, could still be improved. As shown in Figure 11f, sensors 9, 14, and 16 should, in principle, also trigger alerts as the prevailing winds are directed toward them. Conversely, as expected, sensors such as 21, 23, 24, and several others remain unaffected in all models (see Figure 8), since the wind carries the CO_2_ plume in the opposite direction.

## 4. Conclusions

This paper introduces a new paradigm of early warning systems for detecting forest fires using a network of CO_2_ sensors, a data acquisition system, and AI techniques to identify anomalies automatically. The performance of the CO_2_ sensor network was tested in a real scenario with an experiment in situ with a controlled fire. The network comprised 44 sensors located at different distances from the ignition point (from 10 m to 80 m). Three AI models were developed based on AutoEncoder and LSTM to automatically detect CO_2_ anomalies. To assess their performance, the models were compared to a simple NO-AI model, which used the maximum CO_2_ concentration recorded in normal conditions (i.e., no fire) as a threshold, assuming that during the fire experiment such a concentration would be higher.

AI techniques significantly increased the number of sensors detecting alerts during the fire experiment (26, 22, and 19 out of 44 for LSTM, AE CO_2_, and AE ΔCO_2_, respectively, vs. 16 out of 44 obtained by the NO-AI model) still avoiding false alarms (i.e., sensors upwind of the fire and not affected by it were not activated). These techniques also permitted more timely alerts for several sensors, achieving a more efficient and reliable early warning system. In particular, the LSTM model was able to extract informative content from the CO_2_ concentration measurements of the sensors that, compared to a simple threshold method like NO-AI, (i) was able to activate more than a 50% more sensors; (ii) activated the sensors with better timing, thus favoring the prompt intervention by the authorities in charge; and (iii) flexibly and reliably tracked the space-time evolution of the potential fire front propagation direction, adapting to the change in wind direction. These features, which already emerged when using moderate-cost passive sensors, are expected to be further improved with higher-quality sensors.

Future outlooks include implementing such a system at a larger scale, with a higher number of sensors but located at a greater distance from each other, and considering other atmospheric variables monitored, such as temperature and humidity. Other future developments will regard the integration of this point-based detection mode with other classical forest fire detection technologies based on RGB images and AI techniques. Finally, all these systems will be integrated into an all-embracing platform that will also incorporate fire propagation models and fire risk evaluation, with AI-based techniques [32], to obtain predictions about the space-time evolution of the fires automatically detected by the system.

## Figures and Tables

**Table 1:** An overview of previous studies on wildfire analysis and detection. RS: remote sensing; GB: ground-based.

Reference	Data Source	AI Methotodogy
[15]	Aerial RS	CNN
[16]	Aerial RS	MLP and Support Vector Machine
[17]	Aerial and GB RS	Swin Transformer
[18]	GB RS dataset	CNN using transfer learning and augmentation
[19]	GB RS dataset	CNN with a novel activation function
[20]	GB RS network	CNN
[21]	Sensor network	U-Convolutional LSTM (ULSTM)

**Table 2:** Total number of alerts provided by the different models during the experiment in each sensor alerted. The first column indicates the sensor ID. AE CO_2_ refers to the AutoEncoder CO_2_ model, AE ΔCO_2_ refers to the AutoEncoder ΔCO_2_ model, LSTM represents the Long Short-Term Memory model, and the last column is the NO-AI model.

ID	AE CO_2_	AE ΔCO_2_	LSTM	NO-AI
1	**5**	**4**	**7**	**4**
2	**2**	**6**	**3**	**1**
3	**6**	**6**	**5**	**6**
4	**1**	**4**	**2**	**1**
5	0	0	**1**	0
6	**2**	**1**	**4**	**1**
7	**1**	**1**	**2**	**1**
8	**1**	**1**	**1**	0
10	**1**	0	**1**	0
11	**15**	**23**	**15**	**36**
12	**6**	**2**	**7**	**5**
13	**1**	0	**2**	0
14	**1**	0	**1**	**1**
15	0	**1**	**1**	0
17	**21**	**18**	**18**	**32**
18	**16**	**17**	**14**	**31**
19	**1**	0	**1**	**1**
26	**1**	**1**	**1**	0
27	**1**	**3**	**2**	**1**
28	**4**	**1**	**3**	**4**
29	0	0	**1**	0
30	**2**	**2**	**2**	**1**
31	**3**	**4**	**4**	**1**
36	**1**	**1**	**1**	0
43	**1**	**1**	**1**	0
44	0	0	**1**	0

**Table 3:** Ratio between the distance of the sensors from the ignition point to the time interval of the first alert provided by the four different models. When, for a given sensor, a single model performs better than all the others, the value of the ratio is bolded. The first column indicates the sensor ID, the second column AE CO_2_ refers to the AutoEncoder CO_2_ model, the third column AE ΔCO_2_ refers to AutoEncoder ΔCO_2_ model, the fourth column LSTM represents Long Short-Term Memory model, and the last column is the NO-AI model.

ID	AE CO_2_	AE ΔCO_2_	LSTM	NO-AI
1	0.29	0.29	**0.50**	0.29
2	0.29	0.50	0.50	0.29
3	0.29	0.29	0.29	0.29
4	0.10	0.29	0.29	0.10
5	-	-	0.12	-
6	0.11	0.11	**0.50**	0.11
7	0.25	0.25	0.25	0.25
8	0.25	0.25	0.25	-
10	0.25	-	0.25	-
11	0.67	0.67	0.67	0.67
12	0.29	0.29	**1.00**	0.29
13	0.29	-	**0.50**	
14	0.25	-	0.25	0.25
15	-	0.25	0.25	-
17	0.50	0.67	0.67	0.67
18	0.40	0.50	0.50	0.50
19	0.05	-	0.05	0.05
26	0.05	0.05	0.05	-
27	0.07	0.15	0.15	0.07
28	0.07	0.04	**0.08**	0.07
29	-	-	0.40	-
30	0.25	0.25	0.25	0.25
31	0.11	0.12	0.12	0.05
36	0.07	0.07	0.07	-
43	0.25	0.25	0.25	-
44	-	-	0.05	-

## Data Availability

The datasets presented in this article are not readily available because they are part of ongoing studies. Requests to access the datasets should be directed to the corresponding author, Alessio De Rango, at alessio.derango@unical.it.

## Supplementary Materials

The following supporting information can be downloaded at: https://www.mdpi.com/article/10.3390/s25072012/s1, Video S1: Thermal and RGB video recorded during the fire experiment.

## Abbreviations

The following abbreviations are used in this manuscript:

AE CO_2_	AutoEncoder CO_2_
AE ΔCO_2_	AutoEncoder ΔCO_2_
LSTM	Long Short-Term Memory
NO-AI	Allert system without AI techniques
GB	Ground-based
RS	Remote sensing

## Appendix A

**Table A1:** CO_2_ concentration (ppm) statistics recorded during the 5 days before the fire experiment (training period) and the fire experiment period on the 44 sensors.

ID	Training Period						Fire Experiment Period					
	Min	1st Quart	Mean	Median	3rd Quart	Max	Min	1st Quart	Mean	Median	3rd Quart	Max
1	430	468	476	475	484	516	454	465	476	470	480	571
2	391	420	428	427	435	462	405	417	422	422	427	469
3	422	459	468	469	478	505	441	455	467	461	469	603
4	426	447	455	455	463	492	432	446	455	451	459	544
5	470	499	509	508	519	552	484	498	503	503	507	529
6	463	497	504	504	511	543	477	494	504	501	511	583
7	423	452	459	459	466	489	443	452	459	458	464	502
8	453	485	493	493	500	530	477	488	493	493	498	530
9	465	493	502	503	511	538	478	490	494	495	498	514
10	385	412	418	419	424	447	392	407	413	413	417	445
11	404	427	434	435	442	473	413	425	483	438	504	949
12	413	452	461	462	469	495	437	448	458	454	461	518
13	409	444	451	453	459	485	430	441	447	446	451	481
14	402	440	468	481	489	516	465	474	479	480	483	518
15	409	437	443	444	450	478	424	436	440	439	442	470
16	399	428	435	437	443	464	405	422	427	427	431	445
17	423	464	475	477	486	515	448	465	517	480	541	759
18	405	437	444	444	451	480	426	436	481	446	493	886
19	414	447	455	455	464	494	433	440	446	444	449	496
20	410	435	445	445	453	481	421	428	432	432	437	451
21	424	445	453	453	461	483	430	442	446	446	450	463
22	396	422	428	428	435	457	408	417	421	420	423	438
23	402	430	437	437	444	467	416	425	429	429	433	445
24	424	454	462	463	471	498	438	449	453	453	457	472
25	413	449	457	458	465	490	430	444	448	448	451	464
26	393	427	434	434	441	465	413	421	425	424	428	464
27	406	431	438	439	445	472	419	427	433	431	437	493
28	392	429	436	436	442	470	420	428	437	434	439	487
29	385	416	422	423	429	455	400	412	419	417	423	448
30	387	416	422	422	428	452	393	412	418	418	422	461
31	454	479	490	489	500	532	465	476	484	482	488	538
32	409	447	456	456	465	495	435	443	447	447	451	461
33	409	440	448	449	456	483	422	433	439	440	443	454
34	395	425	434	435	442	473	402	416	419	419	422	441
35	395	426	433	435	441	465	413	425	427	427	430	445
36	420	447	453	454	459	482	430	440	444	443	448	475
37	394	433	440	440	446	469	420	433	437	437	440	459
38	407	433	440	441	447	469	417	430	434	433	438	453
39	416	446	452	453	459	482	434	443	446	446	450	458
40	453	478	485	485	492	528	470	481	486	486	490	506
41	413	453	459	459	465	486	441	453	455	455	458	467
42	424	465	474	474	483	513	444	456	460	461	466	478
43	438	461	466	465	470	490	450	459	462	462	465	485
44	384	417	423	423	428	449	403	418	422	422	426	441
